# Is butyrate a natural alternative to dexamethasone in the management of CoVID-19?

**DOI:** 10.12688/f1000research.51786.1

**Published:** 2021-04-06

**Authors:** Nithin K. K, Prakash Patil, Satheesh Kumar Bhandary, Vikram Haridas, Suchetha Kumari N, Sarathkumar E, Praveenkumar Shetty

**Affiliations:** 1 Division of Proteomics and Cancer Biology, Nitte University Center for Science Education and Research, Nitte (Deemed to be University), Mangaluru, Karnataka, 575018, India; 2 Central Research Laboratory, K S Hegde Medical Academy, Nitte (Deemed to be University), Mangaluru, Karnataka, 575018, India; 3 Department of ENT, Justice K S Hegde Charitable Hospital, Nitte (Deemed to be University), Mangaluru, Karnataka, 575018, India; 4 Arthritis Superspeciality Center, Hublic, Karnataka, 580020, India; 5 Department of Biochemistry/Central Research Laboratory, K S Hegde Medical Academy, Nitte (Deemed to be University), Mangaluru, Karnataka, 575018, India

**Keywords:** Butyrate, Butyric acid, CoVID-19, Cytokine storm, Dexamethasone, Gut microbiota, Hyperinflammation, Probiotics

## Abstract

Coronavirus disease 2019 (CoVID-19) caused by Severe Acute Respiratory Syndrome Coronavirus 2 has affected more than 100 million lives. Severe CoVID-19 infection may lead to acute respiratory distress syndrome and death of the patient, and is associated with hyperinflammation and cytokine storm. The broad spectrum immunosuppressant corticosteroid, dexamethasone, is being used to manage the cytokine storm and hyperinflammation in CoVID-19 patients. However, the extensive use of corticosteroids leads to serious adverse events and disruption of the gut-lung axis. Various micronutrients and probiotic supplementations are known to aid in the reduction of hyperinflammation and restoration of gut microbiota. The attenuation of the deleterious immune response and hyperinflammation could be mediated by short chain fatty acids produced by the gut microbiota. Butyric acid, the most extensively studied short chain fatty acid, is known for its anti-inflammatory properties. Additionally, butyric acid has been shown to ameliorate hyperinflammation and reduce oxidative stress in various pathologies, including respiratory viral infections. In this review, the potential anti-inflammatory effects of butyric acid that aid in cytokine storm depletion, and its usefulness in effective management of critical illness related to CoVID-19 have been discussed.

## Introduction

Severe acute respiratory syndrome coronavirus 2 (SARS-CoV-2) is the causative factor for the Coronavirus disease 2019 (CoVID-19) ^1^. SARS-CoV-2 has affected nearly 100 million people around the globe ^2^. SARS-CoV-2 enters the host by binding to its receptor, angiotensin converting enzyme 2 (ACE2), which is expressed mainly in the lungs and intestine ^3– 5^. Upon infection, SARS-CoV-2 causes mild to severe inflammation, which disrupts homeostasis and the integrity of infected organs ^6, 7^. Furthermore, severe infection of CoVID-19 results in systemic inflammation, thrombosis, acute respiratory distress syndrome (ARDS) and multiple organ failure, which may lead to death ^8– 10^. The corticosteroid immunosuppressant, dexamethasone, which attenuates hyperinflammation and cytokine storm, is being used to treat seriously ill CoVID-19 patients and has been found to improve survival in hospitalised patients ^11, 12^. However, the prolonged usage of dexamethasone causes serious adverse effects and gut dysbiosis ^13, 14^. Besides, the hyperinflammation and thrombotic complications associated with CoVID-19 can also be alleviated by various nutrients including vitamins, polyunsaturated fatty acids, minerals, and even amino acids ^15– 20^. The growing number of studies indicate the potential role of nutritional supplement, probiotics, and gut microbiome in mitigating the inflammation and in preventing viral infections including respiratory viral infections ^21^. Alterations of the gut microbiome has been observed during SARS-CoV-2 infection, which significantly reduces the abundance of beneficial microbiome and its metabolites, such as short chain fatty acids (SCFAs) including butyric acid ^22– 24^.

Butyric acid or butyrate can act primarily as an anti-inflammatory molecule and various studies have reported its role in mitigating hyperinflammation via several mechanisms ^25– 27^. For the past several years, our group has worked on the role of proinflammatory regulators in the pathogenesis of various inflammatory disorders and identified that the role of histone deacetylase (HDAC) inhibitor in activating anti-inflammatory molecules. Further, this leads to the simultaneous down regulation of proinflammatory membrane receptors, downstream signalling molecules and respective cytokines, resulting in inflammatory homeostasis. Our *in vitro* preliminary experiments using various cell lines have revealed that the molecular mechanism of butyrate in neutralising inflammatory devastation, induction of anti-inflammatory molecular expression and its translocation to the site of action, is almost similar to dexamethasone ^28^. Consequently, we hypothesise if the SCFA, butyric acid, a HDAC inhibitor, which is synthesized by the gut microbiota, could have strong anti-inflammatory functions with anti-fibrotic properties. Therefore, this article reviews the anti-inflammatory properties of butyric acid or butyrate and its associated molecular pathways involved in controlling the cytokine storm and hyperinflammation associated with SARS-CoV-2 infection. Based on the various positive reports, we presume that butyric acid possesses potent anti-inflammatory activity, which suggests it as an alternative to dexamethasone for the preventive management of primary and secondary complications related to CoVID-19.

## Coronavirus disease 2019

The CoVID-19 pandemic is caused by SARS-Cov-2, which belongs to genera β-coronaviruses and is the seventh known coronavirus to infect humans ^4^. Spike protein of SARS-CoV-2 binds to ACE2, a type I membrane protein ^29^ expressed in the lung, heart, kidney, and intestine ^3, 30, 31^. The majority of SARS-CoV-2 infected cases present with mild symptoms like dry cough, sore throat, fatigue and fever ^9, 10, 32^. Less common symptoms such as myalgia, expectoration, pharyngalgia, dizziness, nausea, headache, haemoptysis, diarrhoea, abdominal pain, and vomiting have also been reported. Lymphocytopenia along with elevated expression of C-reactive proteins (CRP) and inflammatory cytokines are also common ^9, 10, 32, 33^. Infection can progress into severe disease with dyspnoea, grinding glass-like abnormalities and patchy consolidation areas in lungs observed upon imaging; viral pneumonia usually appears after 2–3 weeks of infection ^9, 10, 32, 33^. However, some patients have developed organ failure, septic shock, myocarditis, acute cardiac injury, arrhythmia, pulmonary oedema, severe pneumonia, acute kidney injury, and ARDS. Inflammation, oxidative stress, and fibrosis associated with CoVID-19 is perhaps partially mediated by angiotensin II (AngII), a substrate for ACE2, which degrades it to anti-inflammatory angiotensin (1–7). The accumulation of AngII results in hyperinflammation induced by nucleotide-binding oligomerization domain (NOD) like receptors family pyrin domain-containing 3 (NLRP3) inflammasome and nuclear factor kappa B (NF-κB) activation ^34^. Disrupted immune response in CoVID-19 is further characterized by decreased expression of human leukocyte antigen D related (HLA-DR) on CD(cluster of differentiation)14 monocytes, accompanied by decrease in number of CD4 and CD19 lymphocytes, and natural killer cells along with the continuous production of proinflammatory tumour necrosis factor (TNF)-α and interleukin (IL)-6 secreted by circulating monocytes, subsequently leading to cytokine storm and hyperinflammation ^7^. Hypercytokinemia and hyperinflammation associated with CoVID-19 results in acute lung injury, ARDS and death of the patients ^8, 35, 36^. Furthermore, the SARS-CoV-2 infection may lead to liver injury and its dysfunction, and dysbiosis in the gut, where high expression of ACE2 is observed. Myocardial damage by interaction of SARS-CoV-2 with ACE2 expressed in cardiac pericytes has also been observed ^37^. In addition, the high complication of disseminated intravascular coagulation is known to be associated with the severe form of CoVID-19 ^38^.

SARS-CoV2 infection significantly alters gut microbiota, increasing the number of opportunistic pathogens such as *Clostridium hathewayi*, *Actinomyces viscosus*, *Bacteroides nordii, Streptococcus, Rothia, Erysipelatoclostridium* and *Veillonella* along with significant reduction in beneficial bacteria such as *Lachnospiraceae bacterium* 5_1_63FAA, *Eubacterium rectale*, *Ruminococcus obeum*, *Fusicatenibacter*, *Eubacterium hallii*, *Anaerostipes*, *Agathobacter*, *Roseburia*, *Dorea formicigenerans, Clostridium butyricum, Clostridium leptum and Faecalibacterium prausnitzii,* which includes butyric acid producing bacteria (BPB). Abundance of BPB is negatively correlated with inflammatory and thrombosis markers including CRP, Procalcitonin and D-dimer *.* However, the plethora of opportunistic *Coprobacillus* species, *Clostridium ramosum* and *C. hathewayi* are positively associated with the CoVID-19 severity, but beneficial species *Alistipes onderdonkii* and *Faecalibacterium prausnitzii* show negative correlation. In addition, the probiotic bacteria, *Lactobacillus* and *Bifidobacterium* are also decreased in CoVID-19 patients ^22, 24, 39^. The resulting gut dysbiosis may lead to aberrant inflammation and increased severity of CoVID-19 due to the disruption in the gut-lung axis ^34, 39– 41^. The reduction in BPB may impact lung inflammation and subsequent injury associated with CoVID-19 ^42, 43^.

## Nutrients in mitigating the Covid-19 pathogenesis

Nutrition and nutrients play a vital role in enhancing immune response along with reduction of inflammation and oxidative stress ^44– 46^. Better nutritional status of CoVID-19 patients is associated with less adverse outcomes ^18, 47– 52^. Vitamin D is involved in reducing respiratory infections, such as influenza, and a reduced plasma 25-hydroxyvitamin D (25(OH)D) concentration in SARS-CoV-2 patients has been observed ^53^. Moreover, people with vitamin D deficiency are at higher risk of getting infected with SARS-CoV-2 ^54, 55^. Co-supplementation of vitamin D along with glutathione precursor L-cysteine significantly increases serum 25(OH)D levels and augments vitamin D regulatory gene expression, which in turn reduces the oxidative stress and inflammatory responses in CoVID-19 patients ^56^. Vitamin D supplementation in SARS-CoV-2 infected patients attenuates the production of proinflammatory cytokines like Interferon (IFN)-γ, IL-6, IL-2 and TNF-α by inhibiting NF-κB and other pathways ^57– 59^. CoVID-19 associated inflammatory signalling pathways including NF-κB, Mitogen-Activated Protein Kinase (MAPK) and phosphatidylinositol 3-kinase/ protein kinase B (PI3K/AKT) and innate immune response pathways, such as Toll-like signalling and NOD-like signalling modulation and regulation can be mediated by the combination of curcumin, vitamin C, and glycyrrhizic acid ^60^. Vitamin C has been known to improve the immune condition by enhancing differentiation and proliferation of B- and T-cells, but severe vitamin C deficiency is associated with pneumonia and respiratory tract infections ^61^. Intravenous administration of vitamin C can significantly decrease IL-6 levels ^62, 63^. Glycyrrhizic acid and curcumin exhibits anti-viral, anti-inflammation, anti-cancer, and immune system benefits ^60^. The combination of vitamin D/magnesium/vitamin B12 significantly reduced the subsequent need for oxygen therapy and/or intensive care support in older CoVID-19 patients ^57^. Vitamin B12 is crucial in maintaining the healthy gut microbiome which plays a vital role in immune responses ^57^. Fat soluble vitamin E acts as an antioxidant that scavenges Reactive Oxygen species (ROS) and inhibits devastating effects of hyperinflammation ^64^. Moreover, the supplementation of vitamin E stimulates T cell function and confers protection against upper respiratory infections ^65^.

Selenium is one of the key micronutrients known to positively impact CoVID-19 patient recovery ^66, 67^. Selenium status regulates the expression of glutathione peroxidase 1 (GPX1), a cytosolic selenoenzyme known for its antioxidative properties. The antioxidant enzyme GPX1 mitigates the production of ROS and further leading to mutations in the viral genome ^68^. In addition, attenuating ROS also helps in the inhibition of proinflammatory NF-κB activation and further nuclear translocation ^69^. Severe endothelial injury and widespread pulmonary micro thromboses are accompanied with platelet activation and aggregation in patients with severe CoVID-19 manifestations. The synthetic Rupatadine (histamine1 receptor antagonist) and natural flavonoids with anti-inflammatory properties are known to inhibit the platelet activating factor ^70^. Elderly individuals with deficiency of nutrients, such as vitamin C, vitamin D, calcium, folate, and zinc are prone to increase severity of SARS-CoV-2 infection ^71^. Folic acid may inhibit furin protease and inactivates chymotrypsin-like protease (3CL ^pro^) ^72^. Zinc (Zn ^2+^) deficiency contributes to impaired cell mediated immune response and increased susceptibility to various infections. However, increased intracellular levels of Zn ^2+^ disrupt viral RNA replication including SARS-CoV-2, where Zn ^2+^ inhibits RNA (Ribonucleic acid) dependent RNA polymerase (RdRp) elongation and template binding ^73^. Among CoVID-19 patients, iron deficiency is strongly associated with increased inflammation and longer stay in hospitals ^74^.

Health beneficial compounds, including minerals, antioxidants, phytochemicals, vitamins, and minerals present in fruits and vegetables, can exert antioxidative, anti-inflammatory and antiviral effects during various non-infectious and infectious disease ^71^. Alliin, an S-allyl cysteine sulfoxide compound present in garlic has shown to have inhibitory action on 3CL ^pro^, a protease that plays a vital role in SARS-CoV-2 replication ^75^. Salvianolic acid A and curcumin have the potential to bind to 3CL ^pro ^ with greater affinity ^76^. Resveratrol acts as an anti-inflammatory molecule that inhibits the NFκB pathway and thereby reduces circulatory cytokines, such as IL-6 and TNF-α levels, which are observed in severe SARS-CoV-2 infection ^77^. Sea cucumber ( *Stichopus japonicus*) derived sulphated polysaccharide showed significant anti-viral activity against SARS-CoV-2 infection ^78^. Omega-3 polyunsaturated fatty acids, including eicosapentaenoic acid and docosahexaenoic acid have been shown to exhibit anti-inflammatory effects by downregulation of the NF-κB pathway ^71, 79, 80^. Free fatty acids such as oleic acid, arachidonic acid and linoleic acid have shown antiviral activity at micromolar concentrations ^81^. Dietary fibre intake alters the intestinal microflora and enhances relative proportion of SCFAs, which exhibit anti-inflammatory properties through fatty acid receptors like G-protein-coupled receptor (GPCR) 41 and 43 ^82– 84^.

## Probiotics: suppressors of respiratory tract infections and inflammation

Probiotics are living microorganisms that provide health benefits to the host upon administration at appropriate doses ^85^. Probiotics exert a wide range of beneficial effects such as host microbiome balancing, stimulation of immune system, enhancement of intestinal barrier function or inhibiting pathogens by direct interactions ^40, 46, 86, 87^ ( Table 1). Several microorganisms belonging to the family of *Enterococcus* species ( *E. fecalis, E. faecium*), *Bifidobacterium* species ( *B. bifidum, B. longum, B. lactis*), *Lactobacillus* species ( *L. acidophilus, L. casei, L. rhamnosus*), and *Saccharomyces* ( *S. boulardii, S. cerevisiae*) are considered as probiotics ^40^. Probiotic supplementation causes significant reduction in the incidence of oral and respiratory tract infections ^88, 89^. Dietary supplementation of cow’s milk and fermented rice with *L. paracasei* CBA L74 helps in prevention of common infectious disease including upper respiratory tract infections in children ^90^. Daily intake of fermented milk containing probiotic *L. casei* strain ‘Shirota’ has been shown to reduce the incidence and duration of respiratory tract infections in healthy middle aged office workers and young children via modulation of the immune system ^91, 92^. Daily ingestion of the probiotic *L. paracasei* ST11 can reduce the degree of virus replication and dissemination thereby attenuating lung inflammation and subsequent death in mice infected with vaccinia virus ^95^. *L. gasseri* SBT2055 exhibits antiviral activity against human respiratory syncytial virus (RSV) by silencing SWI2/SNF2-related cAMP Response Element-Binding Protein (CREB)-binding protein activator protein, which is involved in RSV replication. *L. gasseri* SBT2055 reduced the expression of proinflammatory cytokines in lungs upon RSV infection ^96^. CC chemokine receptor 2 acts as a receptor for monocyte chemoattractant protein-1 (MCP-1), which induces increased lung inflammation and subsequently decreases survival associated with influenza virus infection. Prophylactic oral administration of heat-killed *E. faecalis* can protect mice from influenza virus infection and subsequent lung inflammation by modulation of MCP-1 production. Alternatively, lipoteichoic acid of *E. faecalis* binds to toll like receptor 2 and exerts antiviral and anti-inflammatory activity during influenza infection ^97^. Oral administration of probiotics *L. paracasei*, *L. gasseri*, and *B. longum* improved immune response and reduced mortality in influenza infected mice ^105^ by reducing the inflammation and oxidative stress associated with it ^106, 107^.

Probiotics, in combination with enteral nutrition, given to post-operative gastric cancer patients aids in increased production of antibodies and reduction of inflammatory cytokines ^108^. ** Oral administration of *L. plantarum* ameliorates intestinal inflammation and lipid metabolism disorders by modulating gut microbiota in turn producing more SCFAs in high-fat diet induced obese mice ^100^. This disrupted enterohepatic immunoregulation, which can be ameliorated by intervention of *Clostridium butyricum* B1 via its metabolite butyric acid ^99^. Probiotic mixture of *Lactobacillus and Bifidobacterium* prevents the non-alcoholic fatty liver disease by suppressing systemic adiposity and inflammation through butyric acid and its receptor GPR109A ^98^. Treatment with probiotic strain *L. acidophilus* DDS-1 upsurges the abundance of beneficial bacteria such as *Lactobacillus* spp *and Akkermansia* spp and also the levels of butyrate, while downregulating the production of inflammatory cytokines IL-6, IL-1β, IL-1α, MCP-1, Macrophage Inflammatory Protein (MIP)-1α, MIP-1β, IL-12 and IFN-γ in aging mice ^101^. *L. paracasei* KW3110 suppresses hyperinflammation via activation of M2 macrophages and exhibit anti-inflammatory effects via suppression of IL-β production and caspase 1 activation by promoting IL-10 production ^103^. Probiotic complex of *L. acidophilus, L. casei, L. fermentum, L. paracasei, Streptococcus thermophilus, Bifidobacterium longum, B. bifidum, B. breve, L. rhamnosus, L. plantarum, L. helveticus,* and *L. salivarius* in combination with zinc and coenzyme Q10 can improve autoimmune arthritis via downregulation of proinflammatory cytokines including IL-6, IL-17 and TNF-α and inhibition of T-helper cell 17 (Th17) cell differentiation ^109– 111^. Oral administration of *B. infantis* suppresses allergic inflammation in lungs by significantly reducing serum levels of Immunoglobulin (Ig)E, IgG1, IL-4 and IL-13 ^102^ *.* Daily administration of *L. plantarum* DR7 for 12 weeks can prevent development of upper respiratory tract infections among young adults through various mechanisms including inhibition of respiratory infection causing bacteria such as *Staphylococcus aureus*, *Streptococcus pneumoniae*, *Streptococcus pyogenes* and *Streptococcus mutans,* stimulation of proinflammatory cytokine production such as IL-10 and IL-4, and enhancement of antioxidant potential of RBC membrane ^93^. Significant reduction in the number of *Bifidobacteria and Lactobacilli* along with increased number of *Escherichia coli* is observed in the gut of children with recurrent respiratory tract infections. Oral probiotic supplement containing *Bifidobacterium infantis, L. acidophilus, E. faecalis and Bacillus cereus* restored the intestinal flora along with reduction in incidence of respiratory tract infections and use of antibiotics ^23^.

**Table 1.** Ameliorative role of probiotics in suppressing respiratory tract infections and hyperinflammation.

Organism	Dose and Duration		Type of study	Outcome	Reference
*S. salivarius K12,* *S. salivarius M18,* *L. reuteri,* *L. sakei,* and *L. paracasei*	First month: 3 tablets/day, Next two months: one tablet/day		double-blind, randomized, placebo- controlled trial	↓ RTIs in paediatric population	88
*L. paracasei* CBA L74	For 3 Months: 5.9 × 10 ^11^ CFU/day dietary product deriving from cow's milk or rice fermentation		double-blind, randomized, placebo- controlled trial	↓ incidence of URTIs in children attending day care or preschool	90
*L. casei* Shirota	For 12 weeks: 1× 10 ^11^ CFU/day		randomized controlled trial	↓ incidence of URTIs in healthy middle aged office workers	91
	For 12 weeks: 65 mL/day fermented milk, containing 10 ^8^ CFU/mL		controlled open trial	↓ acute RTIs in young Vietnamese children	92
*L. plantarum DR7*	For 12 weeks: 1 × 10 ^9^ CFU/day		randomized, double- blind, placebo-controlled study	↓ duration and frequency URTIs ↓ TNF-α and IFN-γ ↓oxidative stress ↑ IL-10 and IL-14	93
*L. gasseri* A5	For 4 weeks: 1 × 10 ^7^ CFU/day		*In vivo* (Female BALB/c and C57BL/6 mice)	↓mite induced allergic inflammation	94
*L. paracasei* ST11	For 9 days: 10 ^8^ CFU/day		*In vivo* study (mice)	↓vaccinia virus replication, dissemination and infection associated lung inflammation	95
*Lactobacillus gasseri* SBT2055	For 24h: 50 μg/ml		*In vitro* (HEp-2 human laryngeal epithelial cells and MLE12 mouse lung epithelial cells)	↓ RSV replication and associated lung inflammation	96
	For 21 days: 2 × 10 ^9^ CFU/day		*In vivo* (mice)		
*E. faecalis* (heat killed)	For 12 days: 8.5 × 10 ^10^ CFU/kg/ day		pre-treatment, *in vivo* (CCR2-deficient and C57BL/6 mice)	↓ monocyte chemoattractant protein-1 in influenza infection	97
Probiotic mixture containing 6 *Lactobacillus* and 3 *Bifidobacterium*	For 16 weeks: 0.6 g/kg/day (6 billion CFU/g)		*In vivo* (male SD rats, 6 weeks old)	↓systemic adiposity and inflammation	98
*C. butyricum* B1	For 8 weeks: 1×10 ^9^ cells/ day		*In vivo* (male C57BL/6 mice)	↓ Non-alcoholic steatohepatitis and inflammation. ↔ enterohepatic immunoregulation	99
*L. plantarum* Y44	For 12 weeks : 4×10 ^7^ CFU/mL/ day or 4×10 ^9^ CFU/mL/day		*In vivo* (C57BL/6 obese mice)	↓intestinal inflammation ↑gut bacteria and SCFAs production	100
*L. acidophilus* DDS-1	3 × 10 ^9^ CFU/g		*In vivo* (C57BL/6 obese mice)	↓proinflammatory cytokine levels ↑gut microbiota and SCFAs	101
*B. infantis* CGMCC313-02	0.2 mL/day (5 × 10 ^10^ CFU/mL)		*In vivo (*Male BALB/c mice)	↓ allergen induced secretion of IgE, IgG1 and proinflammatory cytokines.	102
*L. paracasei* KW3110	1.25–5 μg/mL	For 24 hours	J774A.1 cells	↓ cytokine IL-1β via IL-10 activation and signalling	103
	100 μg/mL		human monocytes		
*S. thermophilus* DSM 32345, *L. acidophilus* DSM 32241, *L. helveticus* DSM 32242, *L. paracasei* DSM 32243, *L. plantarum* DSM 32244, *L. brevis* DSM 27961, *B. lactis* DSM 32246, and *B. lactis* DSM 32247	For 21 days: 2.4×10 ^9^/day in 3 equal doses/day		cohort study	8 – fold decrease in risk of developing respiratory failure associated with CoVID-19.	104

RTIs-Respiratory tract infections; URTIs- Upper respiratory tract infections; CFU-Colony forming unit; RSV-Respiratory syncytial virus; CCR2- C-C chemokine receptor type 2; IL-Interleukin; IFN-Interferon; TNF-Tumour necrosis factor, SCFAs-short chain fatty acids; CoVID19- Coronavirus disease 2019. ↓-Reduce; ↑-Enhance; ↔- Balance.

Exopolysaccharides produced during milk fermentation by probiotic *L. paracasei* acts as a substrate for the gut microbiome. Fermentation of this exopolysaccharide increases the number of beneficial microbiomes belonging to phyla *Firmicutes* and *Lentisphaerae*, accompanied by the decrease in *Actinobacteria*, *Proteobacteria* and *Bacteroidetes*. Fermentation of exopolysaccharide enhances SCFAs production mainly butyric acid ^112^. Aqueous probiotic supplements containing *L. acidophilus* NCIMB 30175, *L. plantarum* NCIMB 30173, *L. rhamnosus* NCIMB 30174 and *E. faecium* NCIMB 30176 induces an increase in butyric acid producing bacteria resulting in increased production of butyric acid exhibiting immunomodulatory activity via downregulation of proinflammatory cytokines such as MCP-1, Chemokine (C-X-C motif) ligand (CXCL)-10 and IL-8 *in vitro* ^113^. Oral administration of multistrain probiotic mixture containing *L. helveticus* DSM 32242, *B. lactis* DSM 32246, *L. paracasei* DSM 32243, *L. plantarum* DSM 32244, *L. brevis* DSM 27961, *L. acidophilus* DSM 32241, *Streptococcus thermophilus* DSM 32345 and *B. lactis* DSM 32247 decreased development of respiratory failure associated with CoVID-19 by 8 times along with reduction in other symptoms such as diarrhoea, fever, asthenia, headache, myalgia, and dyspnoea ^104, 114^. Use of probiotics may restore the healthy gut microbiome in CoVID-19 patients and exhibit antiviral effects through gut-lung axis. The immunomodulatory role of probiotics helps in viral shedding, regulation of hypercytokinemia and associated multiple organ failure in severe CoVID-19 cases ^40, 115– 117^.

## Is butyrate an alternative to dexamethasone?

Dexamethasone is a synthetic corticosteroid that acts as an anti-inflammatory agent, widely affecting innate and acquired immune system via glucocorticoid receptor ^118, 119^. Low dose dexamethasone treatment significantly supresses neutrophil infiltration and subsequent pulmonary inflammation and significantly improves lung function in early phase of ARDS ^120^. Lower respiratory tract transcriptomic profiling of patients with CoVID-19 associated ARDS shows dysregulated immunoregulation and inflammation. This dysregulated immune response can be modulated by dexamethasone ^121^. A short course of dexamethasone significantly reduces CRP levels and accelerates recovery ^122^. Dexamethasone treatment in CoVID-19 patients who were receiving mechanical ventilation support results in lower mortality rate ^123^. Severe CoVID-19 cases have been brought to remission state after 6 mg once a day intravenous administration of dexamethasone ^124^.

Dexamethasone is indicated as a therapeutic option for immune thrombocytopenic purpura associated with CoVID-19 ^124, 125^. Administration of dexamethasone before 30 hours of ARDS onset can significantly reduce the period of mechanical ventilation and mortality ^12^. Dexamethasone provides an excellent protective effect against hypoxia associated with CoVID-19 ^118^. Intravenous dexamethasone treatment for CoVID-19 patients along with standard care significantly decreases the number of ventilator dependent days over 28 days ^11^. High dose pulse therapy of dexamethasone increased the survival rate in CoVID-19 patients presented with hyperinflammation ^126^. However, dexamethasone, a broad spectrum immunosuppressant, inhibits lymphocytes function and prevents macrophage mediated removal of apoptotic cells, which leads to reduced viral shedding and increases subsequent viremia in mild to moderately ill CoVID-19 patients ^124, 127, 128^. Prolonged use of corticosteroids is associated with serious adverse effects such as short-term hyperglycaemia, cataracts, glaucoma, hypertension, psychological effects, weight gain, increased risk of secondary infections and osteoporosis ^13, 129^. Use of such corticosteroids may induce gut dysbiosis ^14^.

Intestinal microflora widely affects host health and alterations in the gut microbiome is correlated with several disease including respiratory disease ^130^. Commensal gut microbiome and its metabolites can modulate host immunity and can also impact on pro inflammatory and immune-regulatory response ^131^. Increased production of microbiome metabolite SCFAs may improve health condition ^132^. Depletion of SCFA production makes mice more susceptible for allergic lung inflammation. Biological effects exerted by SCFAs is dependent mainly on two mechanisms: SCFA mediated (i) activation of GPCRs and (ii) inhibition of HDAC. SCFAs, via HDAC inhibition, positively impacts the functions and numbers of T-helper 1 cells, T-regulatory cells, and Th17 effector cells resulting in reduced inflammatory response in airway diseases ^130^. The short chain fatty acid, butyrate or butyric acid is produced in the colon by anaerobic bacteria such as *Roseburia intestinalis*, *Faecalibacterium prausnitzii, Clostridium butyricum*, *Megasphaera elsdenii, Mitsuokella multiacida, Eubacterium* spp., *Fusobacterium* spp., *Butyrivibrio* spp. *and Eubacterium hallii* ^133^. Butyrate concentration in the colon can reach from 10 to 20 mM and serves as major source of energy for colonocytes. Sodium butyrate supplementation enhances the abundance of beneficial bacteria such as *Coprococcus, Lachnospiraceae, Ruminococcus, Bifidobacteriaceae and Actinobacteria* improving intestinal barrier integrity in obese mice ^134^.

**Figure 1.**
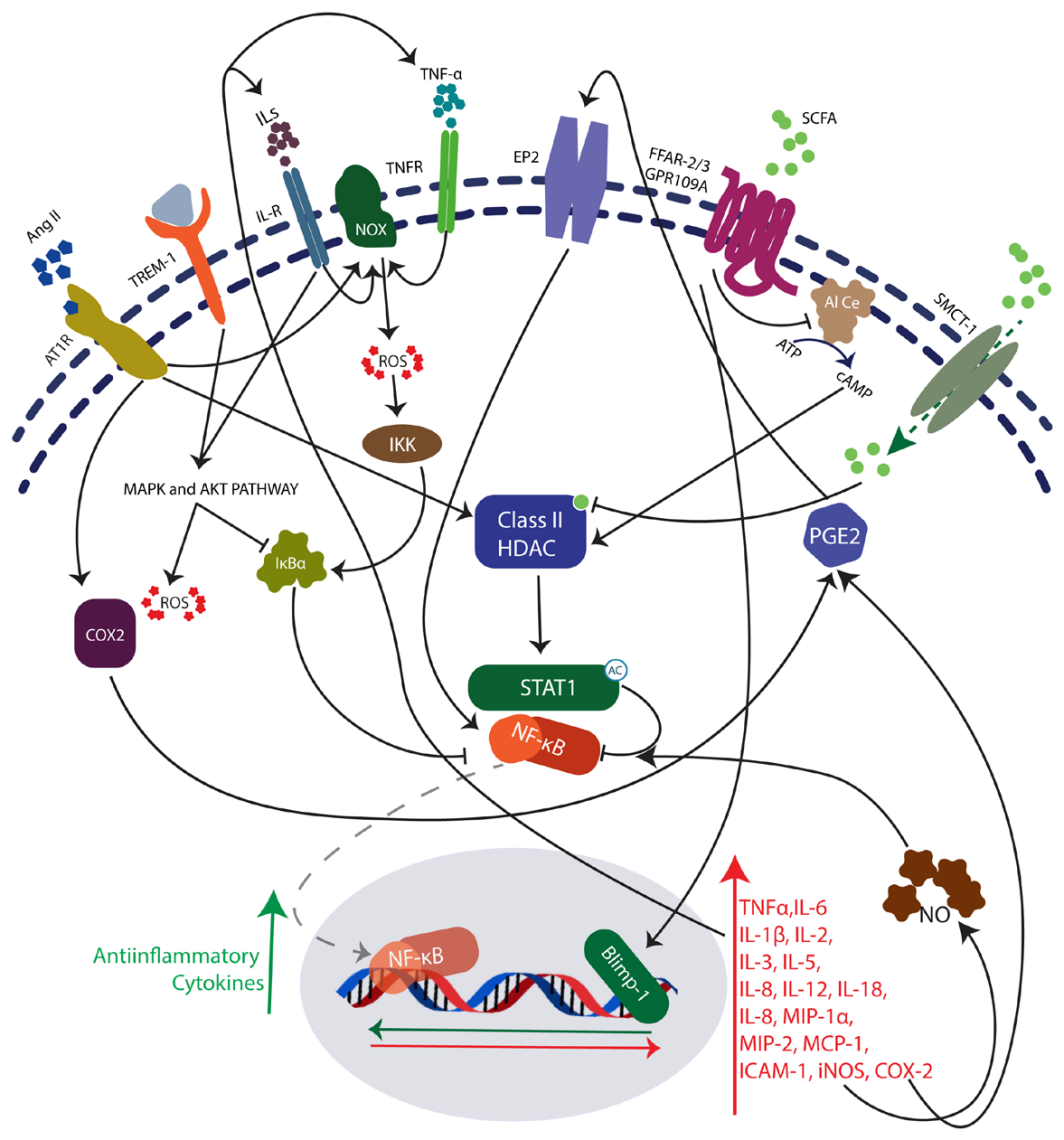
Proinflammatory Angiotensin II, Interleukins, Tumour necrosis factor-α and Triggering receptor expressed on myeloid cells 1 (TREM-1) mediates the activation of Mitogen-activated protein kinase (MAPK), Extracellular signal-regulated kinase (ERK1/2) and Phosphatidylinositol 3-kinase (PI3K)/protein kinase B (AKT) intracellular signalling pathways. The downstream activators of these pathways induces the reactive oxygen species (ROS) generation and transcription factor, NF-κB dependent expression of proinflammatory molecules. HDACs, which deacetylates Signal transducer and activator of transcription 1 (STAT1), and promotes the nuclear translocation and subsequent activity of NF-κB. Target genes of NF-κB, inducible nitric oxide synthase (iNOS) and cyclooxygenase-2 increases the NF-κB activity via positive feedback loop. Histone deacetylase (HDAC) inhibitor, butyrate mediates its effects through GPCRs: Free fatty acid receptors 2/3 and GPCR 109A or by directly binding to HDAC active sites. Inhibition of NF-κB activity by butyrate attenuates inflammation and oxidative stress associated with various pathologies including CoVID-19. Butyrate also activates the transcription factor B lymphocyte-induced maturation protein-1 (BLIMP-1) and enhances the production of anti-inflammatory cytokines.

Primarily, butyric acid exhibits anti-inflammatory and tissue protective function in the large intestine ^135^. Butyric acid is a potential inhibitor of pro-inflammatory molecule NF-κB ^135– 137^ ( Figure 1). Tight junction protein expression in intestinal epithelial cells is also influenced by butyrate mediated regulation ^138^. Butyrate treatment on epithelial colon cells significantly downregulated the proinflammatory molecules including Toll-like receptor (TLR)2, TLR4, IL-6, IL-12A, IL-1β, IL-18, TNF, MAPK13, MAPK10, MAPK3, AKT1, AKT2, AKT3, NF-κB1A, NF-κB1, CXCL1, CXCL2, CXCL3, CXCL6, CXCL8, Chemokine ligands (CCL)2, Serpin peptidase inhibitor, clade A (alpha-1 antiproteinase, antitrypsin), member 1 (SERPINA1), SERPINA2, Colony Stimulating Factor (CSF) 3, Intercellular Adhesion Molecule 1 (ICAM1), Vascular Endothelial Growth Factor A (VEGFA), Major Vault Protein (MVP), Cathelicidin Antimicrobial Peptide (CAMP) and insulin-like growth factor binding protein (IGFBP)3, along with inhibition of proinflammatory pathways, including (i) triggering receptor expressed on myeloid cells (TREM-1) signalling, (ii) production of nitric oxide (NO) and ROS, (iii) high-mobility group box-1 (HMGB1) signalling, (iv) IL-6 signalling, and (v) acute phase response signalling ^25^. Pre-treatment with butyric acid can attenuate heart depression along with reduction in inflammation and oxidative stress associated with septic shock in mice ^139^. Acute lung injury along with ARDS characterized by excessive inflammation can be induced by various factors such as endotoxins, infections, hypoxia and complement activation. Lipopolysaccharide (LPS) induced acute lung injury (ALI) and inflammation can be attenuated by 4-phenyl butyric acid (4-PBA), a derivative of butyric acid and also by sodium butyrate ^26, 140^.

Prophylactic treatment of sodium butyrate significantly reduces myeloperoxidase activity and inflammatory cell infiltration into lungs which is correlated with the inhibition of proinflammatory cytokine, HMGB1 expression and NFκB ^26^. The TLR 4/NF-κB pathway involved in the LPS is targeted by sodium butyrate, which attenuates the LPS induced lung injury ^27^. Hyaluronan ester with butyric acid treatment induces apoptosis in mesangial cells after exposure to oxidative stress and thereby reducing cell proliferation via p38 MAPK pathway ^141^. N-(1-carbamoyl-2-phenyl-ethyl) butyramide (FBA), a butyrate releasing compound, confers protection to mice from colitis induced by dextran sodium sulphate by suppressing neutrophils recruitment and subsequent release of pro-inflammatory molecules mediated by HDAC-9/NF-κB inhibition and peroxisome proliferator-activated receptor gamma (PPAR-γ) upregulation ^142^. Butyrate inhibits IL-13 and IL-15 production by Type 2 innate lymphoid cells. Butyrate downregulates various RNA binding proteins and thereby post transcriptionally downregulating the expression of inflammatory genes ^143^. Sodium butyrate attenuates AngII induced hypertension, cardiac hypertrophy, cardiac fibrosis, and inflammation by inhibiting Cyclooxygenase-2 (COX2)/ Prostaglandin E2 (PGE2) pathway in a HDAC5/ HDAC6 dependent manner ^144^. Butyrate reduces AngII induced endothelial dysfunction ^145^. Sodium butyrate attenuates lung inflammation by promoting forkhead box P3 (FOXP3) expression and suppression of IL-9 expression. Butyrate also reduces the infiltration of proinflammatory Th9 cells and eosinophils into lungs ^146^. Mice treated with butyrate exhibited a significant reduction of inflammatory infiltrates in the airways, tissue, and vascular disruption, and subsequently less haemorrhaging in the lungs induced by influenza infection ^82^. HDAC inhibitor sodium butyrate can suppress ACE2 expression in gut epithelial cells which can help in reducing gastrointestinal symptoms associated with CoVID-19 ^147^.

Pancreatitis and associated fibrosis induced by L-Arginine can be attenuated by sodium butyrate, which reduces collagen deposition and nitric oxide along with inhibition of profibrotic pancreatic stellate cells ^148^. Butyric acid ameliorates bleomycin induced pulmonary fibrosis by attenuating leukocytes infiltration, oxidative stress and NF-κB activation ^149^.

**Figure 2.**
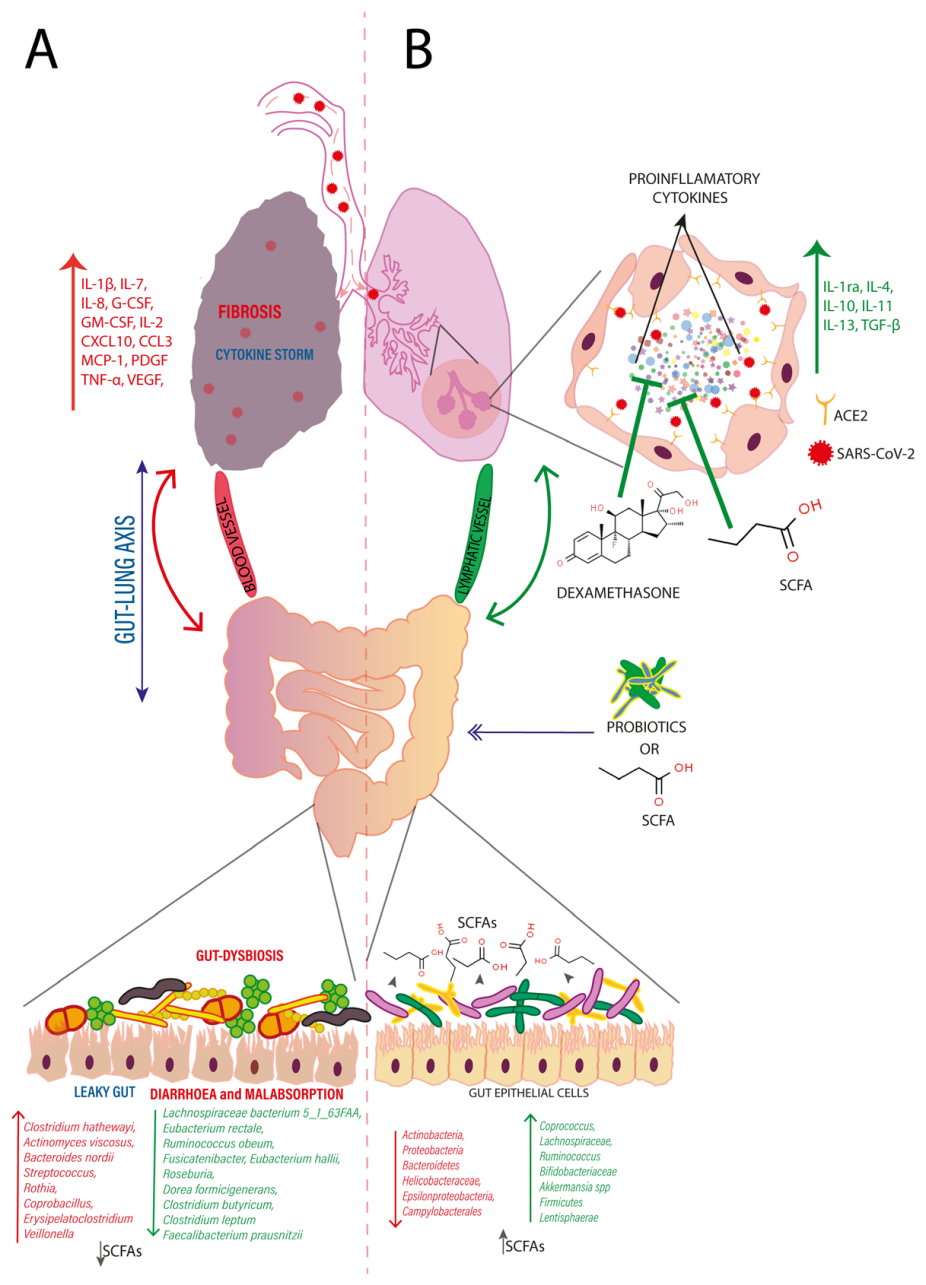
**A**. SARS-CoV-2 transmitted through aerosols reach the lungs via respiratory tract and enters the host cell by binding to its receptor, ACE2 present on the surface of pneumocytes. Followed by endosome mediated internalization, SARS-CoV-2 causes cell injury and subsequent hyperinflammation and cytokine storm, resulting in fibrosis of lungs. These cytokines reach the gut via blood and lymphatic vessels that instigates local inflammation in gut, ushering to leaky gut and gut dysbiosis, resulting in diarrhoea and malabsorption together with reduced production of short chain fatty acids. **B**. Dexamethasone a synthetic broad-spectrum immunosuppressant can inhibit cytokine storm associated with CoVID-19. As an alternative, oral administration of probiotics or gut microbiome metabolite, SCFAs may ameliorate gut inflammation, restore gut integrity, and gut microbiome. This enhances the production of endogenous SCFAs and reaches the lungs via blood and lymphatic vessels, and may inhibit hyperinflammation and cytokine storm along with induction of anti-inflammatory cytokines production which recovers the lung from injury and the acute respiratory distresses associated with CoVID-19.

Consequently, based on the evidence presented, the potential anti-inflammatory and tissue protective effects of butyric acid on lungs and gut, along with its ability to modulate gut microbiome diversity, enhancing production of endogenous butyric acid could be a better preventive approach to manage CoVID-19 over dexamethasone ( Figure 2). However, there is a need for more detailed studies and clinical trials to determine the potency and long-term effect of butyric acid in the preventive management of seriously ill CoVID-19 patients.

## Conclusion

Seriously ill CoVID-19 patients are succumbing to respiratory distress syndrome due to significant hyperinflammation and cytokine storm. A broad-spectrum immunosuppressant, dexamethasone, is widely used to treat such cases. However, the prolonged use of this corticosteroid leads to severe adverse events and disrupted immune responses. There are growing number of advanced research studies in search of an alternative to dexamethasone for the better management of critical CoVID-19 patients. Hence, this review extensively searched for evidence to show the anti-inflammatory properties of butyric acid or butyrate and its associated molecular pathways involved in preventing SARS-CoV-2 infected patients from cytokine storm and hyperinflammation. It has been observed that the SARS-CoV-2 infection significantly decreases butyric acid producing bacteria in the host gut. Further, previous research shows that a histone deacetylase inhibitor, butyric acid has proven to be anti-inflammatory in lung inflammation including inflammation associated with respiratory viral infection. Therefore, based on the various positive reports, we presume that butyric acid possesses potent anti-inflammatory activity, making it a suitable alternative candidate for the preventive management of primary and secondary complications related to CoVID-19.

## Data availabilty

No data is associated with this article.
