# Supplementary material for: Is butyrate a natural alternative to dexamethasone in the management of CoVID-19?
Source: F1000Res. 2021 Apr 6;10:273. [Version 1] doi: 10.12688/f1000research.51786.1 (PMC8108555; doi:10.12688/f1000research.51786.1)
Supplement: Peer review history

# Peer review: Reviewer response for version 1

**DOI:** 10.5256/f1000research.54991.r82768

**Published:** 2021-05-17

**Authors:** Harishkumar Madhyastha

**Affiliations:** 1 Department of Applied Physiology, Faculty of Medicine, University of Miyazaki, Miyazaki, Japan

Abstract can be rewritten, some sentences are incomplete. Various pathologies but only respiratory infection is mentioned.

 Authors mentioned the usefulness of probiotics in management of tissue inflammation, some more clear idea on mechanism to mitigate the inflammation, particularly the viral load is demanded.

 Chemistry of butyrate and dexamethasone and its structure activity relationship in preventing the cytokine storm is necessary.

 Figure 1 - over expressions of anti-inflammatory cytokines is mediated with STAT1 junction proteins and nuclear translocation. Is it possible mention the linking pathway between these?

 Figure 2 - whats is the linker molecules in Gut-Lung axis?

 Future prospective at end would certainly increase the quality of the manuscript.

Is the review written in accessible language?

Yes

Are all factual statements correct and adequately supported by citations?

No

Are the conclusions drawn appropriate in the context of the current research literature?

Yes

Is the topic of the review discussed comprehensively in the context of the current literature?

Yes

Reviewer Expertise:

Signal transduction

I confirm that I have read this submission and believe that I have an appropriate level of expertise to confirm that it is of an acceptable scientific standard.

# Peer review: Reviewer response for version 1

**DOI:** 10.5256/f1000research.54991.r82767

**Published:** 2021-05-07

**Authors:** Stephen O. Mathew

**Affiliations:** 1 Department of Microbiology, Immunology and Genetics, University of North Texas Health Science Center, Fort Worth, TX, USA

The authors have written a comprehensive review on the role of butyrate as a natural alternative to dexamethasone in the management of COVID-19. Various micronutrients and probiotic supplementations are known to aid in the reduction of hyperinflammation and restoration of gut microbiota. The attenuation of the deleterious immune response and hyperinflammation could be mediated by short chain fatty acids (SCFA) produced by the gut microbiota. Butyric acid is well known for its anti-inflammatory properties and ability to reduce oxidative stress and hyperinflammation in many diseases. In this review, the potential anti-inflammatory effects of butyric acid that aid in cytokine storm depletion, and its usefulness in effective management of critical illness related to COVID-19 have been discussed.

 While the review is well written it seems like the title does not fully reflect the content of the review. More than 50% of the review talks about gut microbiome and probiotics and nutrients that play a role in COVID-19 pathogenesis which is not reflected in the title. Only in the second half they talk about the role of butyrate. The title needs to be revised to fully reflect the article.

 There are several new publications that discuss the role of butyrate in COVID-19 pathogenesis which could be added to make this a comprehensive review like J Li *et al.* (2021) ^1^; Sarkar, P *et al.* (2020) ^2^; Wang L *et al.* (2017) ^3^.

 COVID-19 pathogenesis is exacerbated in individuals who have co-morbidities. What is the role of butyrate in co-morbidities? This has not been discussed.

 Innate immune cells play a major role in the COVID-19 pathogenesis. Although the role of butyrate is described in adaptive immune response, its role in innate immunity in the context of COVID-19 pathogenesis is minimally mentioned.

 Some of the molecules like PGE2, ROS-IKK pathway has not been described in Figure 1 legend.

Is the review written in accessible language?

Yes

Are all factual statements correct and adequately supported by citations?

Yes

Are the conclusions drawn appropriate in the context of the current research literature?

Yes

Is the topic of the review discussed comprehensively in the context of the current literature?

Yes

Reviewer Expertise:

Tumor immunology, NK cell biology, innate immunity

I confirm that I have read this submission and believe that I have an appropriate level of expertise to confirm that it is of an acceptable scientific standard.

## References

[1] : Butyrate Regulates COVID-19–Relevant Genes in Gut Epithelial Organoids From Normotensive Rats. *Hypertension*.2021;77(2) : 10.1161/HYPERTENSIONAHA.120.16647 10.1161/HYPERTENSIONAHA.120.16647 PMC781023933439735

[2] : Can microbial SCFA, Butyrate be the alternate Savioragainst COVID-19?. *Curr. Trends Pharm. Res*.2020;7(1) :11-14

[3] : Sodium butyrate suppresses angiotensin II-induced hypertension by inhibition of renal (pro)renin receptor and intrarenal renin-angiotensin system. *J Hypertens*.35(9) : 10.1097/HJH.0000000000001378 1899-1908 10.1097/HJH.0000000000001378 28509726PMC11157961

# Peer review: Reviewer response for version 1

**DOI:** 10.5256/f1000research.54991.r82836

**Published:** 2021-05-06

**Authors:** Mallika Valapala

**Affiliations:** 1 School of Optometry, Indiana University, Bloomington, IN, USA

The review article by Nithin *et al*. discusses whether butyrate can be a natural alternative to dexamethasone in the management of COVID-19. The manuscript is well written and with a clear explanation of purpose. The authors illustrate divergent signaling pathways associated with the pathophysiology of COVID-19. They also point out the issues that dexamethasone has and why butyrate can be an alternative. Listed below are some of my concerns:

1. The authors mention many benefits of butyrate in this review. I suggest the authors should also mention whether there are any side effects of butyrate in specific groups, such as pregnant women and those with underlying conditions. Such a description would enable a more comprehensive assessment of this compound.
2. The authors provided a schematic of pro-inflammatory intracellular signaling pathways and in same schematic describe the mechanism of action of butyrate. There are many signaling pathways in this schematic and readers are easy to get confused. I suggest the authors simplify the schematic or separate it into two different schematics, one for butyrate regulation and another one for the regulation of pro-inflammatory mediators.

Is the review written in accessible language?

Yes

Are all factual statements correct and adequately supported by citations?

No

Are the conclusions drawn appropriate in the context of the current research literature?

Yes

Is the topic of the review discussed comprehensively in the context of the current literature?

Yes

Reviewer Expertise:

Cell biology

I confirm that I have read this submission and believe that I have an appropriate level of expertise to confirm that it is of an acceptable scientific standard.
